# Lipoprotein(a) as Orchestrator of Calcific Aortic Valve Stenosis

**DOI:** 10.3390/biom9120760

**Published:** 2019-11-21

**Authors:** Johan G. Schnitzler, Lubna Ali, Anouk G. Groenen, Yannick Kaiser, Jeffrey Kroon

**Affiliations:** 1 Department of Experimental Vascular Medicine, Amsterdam Cardiovascular Sciences, Amsterdam UMC, University of Amsterdam, Meibergdreef 9, 1105 AZ Amsterdam, The Netherlands; j.g.schnitzler@amsterdamumc.nl (J.G.S.); l.ali@amsterdamumc.nl (L.A.); anoukgroenen@hotmail.com (A.G.G.); 2 Department of Vascular Medicine, Amsterdam Cardiovascular Sciences, Amsterdam UMC, University of Amsterdam, Meibergdreef 9, 1105 AZ Amsterdam, The Netherlands; y.kaiser@amsterdamumc.nl

**Keywords:** aortic valve stenosis, lipoprotein(a), inflammation, calcification, valve interstitial cells

## Abstract

Aortic valve stenosis (AVS) is the most prevalent valvular heart disease in the Western World with exponentially increased incidence with age. If left untreated, the yearly mortality rates increase up to 25%. Currently, no effective pharmacological interventions have been established to treat or prevent AVS. The only treatment modality so far is surgical or transcatheter aortic valve replacement (AVR). Lipoprotein(a) [Lp(a)] has been implicated as a pivotal player in the pathophysiology of calcification of the valves. Patients with elevated levels of Lp(a) have a higher risk of hospitalization or mortality due to the presence of AVS. Multiple studies indicated Lp(a) as a likely causal and independent risk factor for AVS. This review discusses the most important findings and mechanisms related to Lp(a) and AVS in detail. During the progression of AVS, Lp(a) enters the aortic valve tissue at damaged sites of the valves. Subsequently, autotaxin converts lysophosphatidylcholine in lysophosphatidic acid (LysoPA) which in turn acts as a ligand for the LysoPA receptor. This triggers a nuclear factor-κB cascade leading to increased transcripts of interleukin 6, bone morphogenetic protein 2, and runt-related transcription factor 2. This progresses to the actual calcification of the valves through production of alkaline phosphatase and calcium depositions. Furthermore, this review briefly mentions potentially interesting therapies that may play a role in the treatment or prevention of AVS in the near future.

## 1. Introduction

Aortic valve stenosis (AVS) is the most prevalent valvular disease in the Western world. Its prevalence increases exponentially with age, affecting 8% of adults over 80 years of age [1,2]. The early stages of AVS are predominantly asymptomatic [3], but once impaired leaflet movement leads to significant left ventricular outflow obstruction, patients develop angina, heart failure, and syncope [4]. Clinically manifested AVS reaches yearly untreated mortality rates up to 25%. Despite this detrimental prognosis, effective pharmacological interventions to treat or prevent AVS are not available. Currently, patients only become eligible for surgical or transcatheter aortic valve replacement (AVR) once they develop severe AVS, due to considerable periprocedural morbidity and mortality [5,6]. AVS was traditionally considered a passive degenerative disease, caused by continuous wear and tear of the valve, but nowadays it has become evident that the disease is characterized by an active process of inflammation followed by fibro-calcific remodeling of the valve [7,8]. Although there are several well established risk factors for AVS, such as age, bicuspid valve morphology, hypertension, and elevated levels of low-density lipoprotein cholesterol (LDL-C) [9,10], previous randomized trials assessing the effect of statins and angiotensin receptor blockers have unfortunately been unable to show benefit in patients with mild to moderate AVS [11,12,13]. In part, this can be attributed to a different, likely causal risk factor for AVS: Lipoprotein(a) [Lp(a)].

Lp(a) is a low-density-lipoprotein-like particle which is covalently bound to an apolipoprotein(a) [apo(a)] tail [14,15]. A strong association between Lp(a) and AVS was first described in 1995 by Gotoh et al. They reported that amongst 748 middle-aged men and women in a rural village in Japan, the prevalence of aortic valve sclerosis on echocardiography was twofold higher in subjects with Lp(a) levels in the upper quartile (>30 mg/dL), independent of other risk factors [16]. Nowadays, there is an increasing body of evidence indicating that Lp(a) is a causal risk factor for AVS [10,17,18]. Apo(a) is the preferential carrier of oxidized phospholipids (OxPL) in the circulation [19,20], which are considered highly atherogenic and play a pivotal role in Lp(a)-induced AVS [21,22,23].

## 2. Relevance of Lp(a) in AVS

While Lp(a) has been associated with an increased risk for AVS for more than two decades, only two years after the previously described publication of Gotoh, a cross-sectional analysis of 5201 subjects from the Cardiovascular Health Study corroborated the strong association between Lp(a) levels (75th vs. 25th percentile) and echocardiographic AVS [9]. The first longitudinal study which suggested that Lp(a) is a strong risk factor for AVS was conducted in the European Prospective Investigation into Cancer (EPIC)-Norfolk study in 2014. Here, participants in the upper Lp(a) tertile (>19.2 mg/dL) had a higher risk of hospitalization or mortality due to AVS compared with those in the bottom Lp(a) tertile (<7.7 mg/dL; hazard ratio (HR) 1.57; 95% confidence interval, 1.02–2.42). Furthermore, the rs10455872 genetic variant in the *LPA* gene, which is associated with higher Lp(a) levels, increased the risk of AVS (HR of 1.57; 95% confidence interval, 1.10–2.26). These findings indicate that elevated Lp(a) plays a causal role in the development of AVS [18]. In line with these data, analyses from the Copenhagen City Heart Study and Copenhagen General Population Study showed that a 10-fold increase in Lp(a) plasma levels led to an age- and sex-adjusted observational hazard ratio of AVS of 1.4 [10]. Moreover, in patients with heterozygous familial hypercholesterolemia, whose largest risk factor is their lifelong extremely high LDL-C burden, Lp(a) remains predictive of AVS after multivariate analysis, further indicating that Lp(a) is an independent risk factor for AVS [24]. The presence of coronary artery disease (CAD) is a known risk factor for AVS, as impaired ventricular function leads to a more rapid onset of symptoms when the aortic orifice narrows. However, even in patients with established CAD, Lp(a) persists as a risk factor for AVS, implying that in addition to this, Lp(a) affects a different pathway in the pathophysiology of AVS as well [25]. The presence of OxPLs on Lp(a) as a crucial driving factor in the development of cardiovascular disease has previously been described in-depth by Tsimikas et al. [26,27,28], hence it will not be discussed in detail in this review. Lp(a) and its associated OxPLs are linked to AVS progression, not merely because Lp(a)-patients (top tertile > 58.5 mg/dL) with additional increased levels of OxPL-ApoB (reflecting the biological activity of Lp(a)), have faster progression of AVS [29]. Recently, a comprehensive study combined positron emission tomography (PET), computed tomography (CT), and echocardiography to investigate the association between OxPLs, elevated levels of Lp(a), valve calcification activity and AVS progression. This study elegantly showed that both elevated Lp(a) and OxPL-ApoB are independently associated with increased valvular ^18^F-sodium fluoride (NaF) uptake, a measure of micro-calcification predicting AVS progression [30]. During the follow-up visit after one year, patients in the top Lp(a) tertile were characterized by increased aortic valve calcification on the subsequent CT, more rapid echocardiographic progression, and most importantly: An increased incidence of AVR and death [31]. In short, there is strong evidence to support that Lp(a) plays a key role in the development AVS. In the next section, we will delve into the mechanisms behind Lp(a)/OxPL-induced AVS in order to better understand the pathophysiological process.

## 3. Pathophysiology of Lp(a)-Induced AVS 

The pathophysiology of AVS can be divided in two phases: An initiation phase and a propagation phase. The initiation phase of AVS precedes calcification and is similar to the pathophysiology of atherosclerosis [32,33,34,35,36]. In the aortic valve, the initiation phase is ignited by damage to the upper cell layer of the valve comprising of valvular endothelial cells (VEC), a process caused by mechanical, oxidative, or shear stress [37]. In physiological conditions, VECs maintain valvular homeostasis by regulating cell-adhesion, permeability, and paracrine signaling. However, local inflammation and shear stress causes increased valve permeability allowing lipoproteins such as LDL-C and Lp(a) and inflammatory cells (e.g., T-lymphocytes, macrophages, mast cells) to infiltrate the valve. Following infiltration, cells and lipids induce an inflammatory environment within the inner layers of the aortic valve, where valvular interstitial cells (VIC) reside, the most abundant cell population in the aortic valve [38,39,40,41]. Concomitant microcalcifications arise at damaged sites of VECs, which in turn lead to cell death and the release of apoptotic bodies [32,33,42]. These apoptotic bodies facilitate the formation of hydroxyapatite crystals, which contribute to AVS progression by triggering pro-inflammatory cytokine secretion such as IL-1β and IL-6 [40,43]. Furthermore, activated VECs release bone morphogenetic protein 2 (BMP2) which acts as important initiator of calcification [44]. Once inflammation leads to the formation of calcium deposits, the decreased compliance of the aortic valve leads to further mechanical damage, apoptosis, and calcification, inducing a vicious cycle in which calcium begets calcium. Dweck et al. demonstrated this process in humans by utilizing multimodality imaging. ^18^F-NaF uptake in the aortic valve, reflecting the degree of micro-calcifications, shows a stepwise increase for each subsequent disease stage in patients with AVS. Interestingly, valvular ^18^F-fluorodeoxyglucose (^18^F-FDG) uptake, a commonly used tracer to assess arterial wall inflammation, was also increased in patients with AVS, but ^18^F-FDG uptake did not differ much between early and late stage AVS. This indicates that the inflammatory component stays rather stable throughout the disease, whereas calcium deposits are likely to be the main driver of progression during the later stages [45].

The main characteristic of the propagation phase is the differentiation of VICs into an osteoblast-like phenotype. Under healthy conditions, VICs synthesize collagen, elastin, and glycosaminoglycans to provide strength and elasticity and crosstalk with VICs [44,46]. In vitro, Lp(a) incubation of human derived VICs for one week leads to increased gene expression of *IL6*, *BMP2* and *RUNX2*. These osteogenic effects were attenuated once VICs were co-incubated with the E06 monoclonal antibody against OxPLs [31]. The role of OxPLs on Lp(a) was further substantiated using recombinant apo(a) (r-apo(a)) constructs with different lysine binding sites (LBS) to bind OxPLs. Constructs containing a mutation in the LBS lack the ability to bind OxPLs and were unable to increase *IL6*, *BMP2* and *RUNX2* gene expression compared to their r-apo(a) counterparts carrying OxPL. These data support the pivotal role of OxPLs in mediating Lp(a)-induced calcification of VICs [31]. To further unravel this OxPL-mediated calcification, Bouchareb et al. proposed a mechanism by which Lp(a) and its associated OxPLs induce calcification in human VICs through signaling via lysophosphatidic receptor (LPAR). Briefly, the ligand for LPAR is lysophosphatidic acid (LysoPA), which arises upon conversion of lysophosphatidylcholine (LPC) by autotaxin (ATX). ATX, encoded by the *ENPP2* gene is an enzyme that is secreted by various types of cells including VICs. Upon conversion, LysoPA binds to its G coupled-protein receptor LPAR expressed by VICs, initiating an NF-κB-mediated inflammatory cascade. Interestingly, valves from AVS subjects showed a marked increase in both LysoPA and ATX. Detailed immunohistochemical analysis of these valves revealed high presence of ATX colocalizing with OxPLs and apo(a), suggesting the presence of ATX on Lp(a) [47]. Of note, mice lacking LPAR were shown to have reduced bone mass and osteogenesis indicating the importance of LPAR in osteogenesis [48]. Thus, accumulation of Lp(a) in the valve allows ATX to convert LPC to LysoPA and act directly on VICs by binding to LPAR initiating an osteogenic environment. In addition, *ATX* mRNA levels in stenotic aortic valves were associated with *IL6* mRNA expression, a known downstream target of NF-κB [47,49]. The latter was shown in high ATX-containing valves via increased phosphorylation of IKKα (a subunit of NF-κB), indicative of a pro-inflammatory environment [47,49]. In vitro stimulation of human derived VICs with LPC induced secretion of IL-6, which in turn promotes the expression of *RUNX2* and *BMP2*, leading to an osteoblast-like phenotype of VICs [47,50]. In order to further investigate the molecular pathway induced by LPC, Bouchareb et al. inhibited different steps along the expected pathway. Indeed, stimulation of human derived VICs with LPC together with the inhibition of either IκB or BMP2 but also IL-6 silencing hampered mineralization of VICs, showing that the inflammatory and pro-calcific pathways are closely intertwined. Finally, blocking LPAR using ki16425 abolished LPC and LysoPA-mediated induction of IL-6 and reduced Ca^2+^ deposition in vitro [47]. In addition, El Husseini et al. previously reported that IL-6 promotes calcification through Akt-1 signaling. This shows that Lp(a) and its associated OxPLs induce inflammation (i.e., IL-6 secretion by VICs) and thereby contribute to calcification of aortic valves [50]. Additionally, alkaline phosphatase (ALP) plays a crucial role in facilitating mineralization through hydrolysis of pyrophosphate providing inorganic phosphate to fuel mineralization. Several studies have reported that BMP2 regulates the expression of ALP through the MAPK/P38/NF-κB pathway [51,52,53]. In line with this, it has been shown that oxidized LDL initiates mineralization of human VICs through stimulation of biglycan which in turn directly affect the MAPK/p38/NF-κB-pathway through Toll-like receptor 2 signaling [53].

## 4. The Notch and Wnt-Mediated Calcific Regulatory Pathways

Other signaling cascades via which Lp(a) and its associated OxPLs could potentially promote calcification and which predominantly drive osteogenesis are the calcific regulatory pathways NOTCH1 and Wnt/β-catenin. [40,54]. Normally, NOTCH1 signaling prevents calcification through suppression of master osteoblast transcription factor runt-related transcription factor 2 (RUNX2) whereas loss-of-function mutations in NOTCH1 result in upregulation of BMP2 [55,56,57,58,59]. In addition, activation of glycogen synthase kinase-3 (GSK-3β) and β-catenin in the Wnt/β-catenin signaling pathway also results in osteogenic differentiation of VICs via increased production of ALP [60,61]. Interestingly, methylation of NOTCH1 at the promotor side decreases NOTCH1 expression while the Wnt/β-catenin is being activated leading to transcription of the osteogenic genes *RUNX2*, *SOX9*, and *BMP2* suggesting an interplay between the NOTCH1 and Wnt/β-catenin pathway [62]. Together with the activation of these regulatory pathways, the release of pro-inflammatory cytokine IL-6 and pro-osteogenic cytokines (insulin-like growth factor 1 and transforming growth factor-β) is increased, further promoting the osteogenic differentiation of VICs [25,63].

In short, Lp(a) and its associated OxPLs infiltrate the inner layers of the aortic valves to act locally on VIC phenotype. LPC carried by OxPL and Lp(a) is converted to LysoPA by ATX and becomes a ligand for LPAR. Upon binding, an intracellular signaling cascade initiates the activation of NF-κB and potentially via NOTCH1 and Wnt/β-catenin-signaling which in turn leads to increased transcripts of *IL-6*, *RUNX2* and *BMP2*, aggravating calcification of VICs. Inhibition of either IL-6, BMP2 or NF-κB pathway hampered calcification. Additionally, LPAR antagonist also prevented LysoPA induced IL-6 secretion and subsequent mineralization of human VICs.

## 5. Future Perspective

Thus far, there are no medical treatment options attenuating aortic valve inflammation or calcification. First, statins have been widely investigated in AVS, and three randomized controlled trials showed that statin therapy did not attenuate disease progression of AVS, further substantiating calcification as a process irreversible by lipid lowering [11,12,13,64]. In short, Cowell et al. randomized patients to either 80 mg atorvastatin daily or placebo. Here, despite a profound reduction in serum LDL-C (post-treatment levels of 63 ± 23 mg/dL for atorvastatin-treated subjects, versus 130 ± 30 mg/dL in the placebo group), Doppler echocardiography and helical CT of the aortic valve showed no decrease in AVS progression [11]. Next, Rossobø et al. conducted the largest trial in patients with mild-to-moderate AVS, randomizing patients to 40 mg simvastatin plus 10 mg ezetimibe or placebo. After a median follow-up of 52.2 month, their primary outcome (combined aortic valve events and ischemic CV events) occurred in 333 patients in the ezetimibe plus simvastatin group and in 355 patients in the placebo group (HR in ezetimibe plus simvastatin group, 0.96; 95% confidence interval, 0.83 to 1.12; *p* = 0.59), indicating that LDL-C-reduction does not reduce the incidence of events related to AVS [12]. Finally, the ASTRONOMER trial corroborated these results by showing that a daily dose of 40 mg rosuvastatin did not reduce echocardiographic progression in patients with mild to moderate AVS [13]. Possibly counteracting their benefit, statins increase Lp(a) levels, which has been reported by Tsimikas et al. in an individual-participant-data analysis of six statin trials. They assessed Lp(a) levels during statin therapy using a single, well-established assay. Their analysis revealed that the odds ratio of geometric means for statin to placebo was 1.11 (1.07–1.14; ratio > 1 indicates a higher increase in Lp(a) from baseline in statin vs. placebo), indicating that statin treatment increases Lp(a) levels and might be unfit to attenuate AVS, especially in patients with high Lp(a) levels [65].

Another lipid-lowering therapy comprises the use of monoclonal antibodies directed against proprotein convertase subtilisin/kexin type 9 inhibitors (PCSK9). *Pcsk9^−/−^* mice show lower calcification of the valves compared to WT mice, which was further substantiated in vitro *Pcsk9^−/−^* VIC calcification. Human calcified aortic valves showed high expression of PCSK9 compared to non-calcified valves, indicating PCSK9 inhibition might be an interesting therapeutic option to treat AVS [66]. However, this might only be an option for patients with low levels of Lp(a), as evolocumab administration only effectuates small percentage reductions in patients with very high Lp(a) levels [67]. Furthermore, the same study evaluating the effect of evolocumab on arterial wall inflammation in patients with elevated Lp(a) showed no decrease in arterial ^18^F-FDG uptake, suggesting that LDL-C-lowering alone is not sufficient to attenuate Lp(a)-induced inflammation [67].

A major breakthrough in Lp(a)-lowering therapy using antisense oligonucleotides targeting Lp(a) changes the perspective of Lp(a)-patients. IONIS-APO(a)_Rx_-treatment of patients with elevated levels of Lp(a) led to a massive dose-dependent decrease of 66–92% after 36 days whereas OxPL-ApoB and OxPL-apo(a) led to a more moderate decrease of 45% and 25%, respectively [68]. Together these data indicate that lowering Lp(a) levels is promising in order to attenuate the progression of AVS. In particular, OxPL signaling seems to play a pivotal role in AVS since lipid-lowering, other than Lp(a), using statins do not influence progression. 

Therefore, targeting or neutralizing OxPLs might offer a solution which was elegantly shown in in mice with *Ldlr^/−^* background expressing a single-chain variable fragment of E06 (*Ldlr*^−/−^/E06-scFc; which neutralizes OxPL signaling). These mice scored a 49% lower AV pressure gradient (as a measure for AV functioning) compared to their *Ldlr^/−^*-counterparts, lower total calcium content, and all *Ldlr*^−/−^/E06-scFc survived where almost half of the *Ldlr^/−^*-group did not survive [22]. Together with the in vitro OxPL-data of Zheng et al. [31], these data imply a causal relation between OxPL-signaling and the progression of AS and that targeting OxPLs offers an interesting approach to treat the progression of AVS.

Furthermore, anti-inflammatory treatment may serve as an interesting option to treat AVS. Recently it was shown that in AVS-patients who underwent AVR had lower levels of plasma IL-6 after surgery, indicating that neutralizing IL-6 may be beneficial for attenuation of AVS [69]. Monoclonal humanized mouse antibody inhibitor of IL-6 receptor (Tocilizumab; Actemra, Roche AG, Basel, Switserland) antagonizes IL-6 signaling by preventing IL-6 binding with its corresponding receptors which is currently approved to treat rheumatoid arthritis [70]. In vascular smooth muscle cells it was shown that in vitro neutralization of IL-6 using anti-IL-6 antibodies reduced *BMP2* and *RUNX2* expression leading to decreased calcification [71]. Hence, anti-IL-6 treatment would serve as an interesting drug to target AVS progression in patients with elevated levels of Lp(a).

Another interesting strategy for the combat of AVS is the modulation of LPAR signaling. LPAR antagonists have been used in vitro stimulation of VICs with LPC. LPC induced IL-6 cytokine secretion which was prevented by the LPAR antagonists. Furthermore, targeting the LPAR leads to decreased mineralization of VICs [47]. In agreement, mice lacking LPAR display decreased osteoblastic differentiation leading to reduced bone mass [48]. In patients with idiopathic pulmonary fibrosis, LPAR antagonists led to a significant rate of decline in disease progression. Although the LPAR antagonist was well tolerated in most patients, elevations of hepatic transaminases and ALP were detected in some patients [72]. Together these data indicate that antagonizing LPAR signaling may offer an interesting therapy for AVS.

## 6. Conclusions

**Figure 1:**
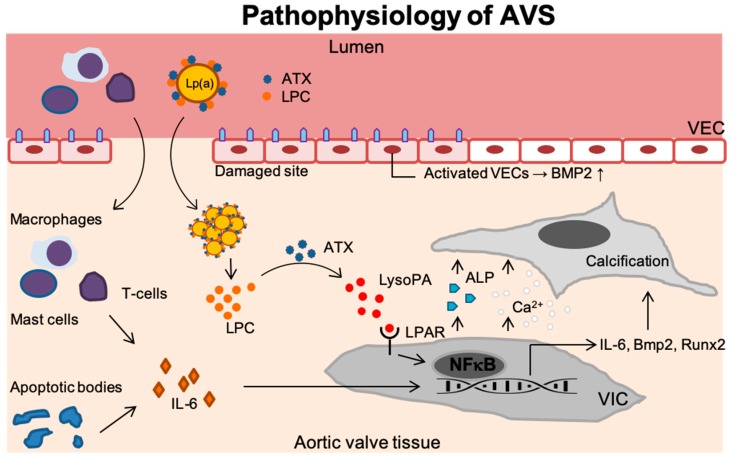
Disease progression in AVS. Upon endothelial damage, an inflammatory environment arises once immune cells (i.e., T-lymphocytes, mast cells and macrophages) enter the valvular tissue and secrete IL-6. In addition, endothelial damage leads activation of VECs which thereby produce BMP2 but also the formation of apoptotic bodies further contributes to inflammation. Lp(a) carrying ATX and LPC accumulates in the valves. ATX then converts LPC into LysoPA which binds the LPAR leading to activation of NF-κB. The activated VICs now increase the *IL6*, *BMP2*, and *RUNX2* transcripts but secrete ALP as well. This leads to increased calcium deposition and calcification of the aortic valve tissue. Lp(a): Lipoprotein(a); ATX: Autotaxin; LPC: Lysophosphatidylcholine; VEC: Valvular endothelial cell; IL-6: Interleukin 6; LysoPA: Lysophosphatidic acid; LPAR: Lysophosphatidic acid receptor; VIC: Valvular interstitial cell; NF-κB,: Nuclear factor -κB; Runx2: Runt-related transcription factor 2; BMP-2: Bone morphogenetic protein-2; Ca^2+^: Calcium; ALP: Alkaline phosphatase.

This review highlights Lp(a) as a highly prevalent and causal risk factor in the development of AVS of which the most important signaling routes are shown in Figure 1. Furthermore, elevated Lp(a) and its associated OxPLs are predictive for faster disease progression in patients with established AVS. Currently, there are no therapeutic options beyond AVR in the end stage of the disease, underscoring the need for novel, earlier interventions to treat AVS. However, with antisense oligonucleotides targeting Lp(a) and neutralizing antibodies against OxPLs, novel therapeutic strategies to combat the initial phase of AVS might be on the horizon.
